# The utility of the Edmonton Obesity Staging System for the prediction of COVID-19 outcomes: a multi-centre study

**DOI:** 10.1038/s41366-021-01017-8

**Published:** 2022-01-01

**Authors:** Marcela Rodríguez-Flores, Eduardo W. Goicochea-Turcott, Leonardo Mancillas-Adame, Nayely Garibay-Nieto, Malaquías López-Cervantes, Mario E. Rojas-Russell, Lilia V. Castro-Porras, Eduardo Gutiérrez-León, Luis F. Campos-Calderón, Karen Pedraza-Escudero, Karina Aguilar-Cuarto, Eréndira Villanueva-Ortega, Joselin Hernández-Ruíz, Guadalupe Guerrero-Avendaño, Sheyla M. Monzalvo-Reyes, Rafael García-Rascón, Israel N. Gil-Velázquez, Dora E. Cortés-Hernández, Marcela Granados-Shiroma, Brenda G. Alvarez-Rodríguez, Martha L. Cabello-Garza, Zaira L. González-Contreras, Esteban Picazo-Palencia, Juana M. Cerda-Arteaga, Héctor R. Pérez-Gómez, Roberto Calva-Rodríguez, Gerardo Sánchez-Rodríguez, Leslie D. Carpio-Vázquez, María A. Dávalos-Herrera, Karla M. Villatoro-de-Pleitez, Melissa D. Suárez-López, María G. Nevárez-Carrillo, Karina Pérez-Alcántara, Roopa Mehta, Edurne Sandoval Diez, Edward W. Gregg

**Affiliations:** 1 National Institute of Medical Sciences and Nutrition Salvador Zubirán, Mexico, CDMX Mexico; 2 General Hospital Dr. Manuel Gea González, Mexico, CDMX Mexico; 3 Nuevo León University Hospital, Mexico, NL Mexico; 4 General Hospital of Mexico Dr. Eduardo Liceaga, Mexico, CDMX Mexico; 5 Public Health Department, Medicine Faculty, UNAM, Mexico, MX Mexico; 6 Faculty of Higher Studies Zaragoza, UNAM, Mexico, MX Mexico; 7 High Specialty Hospital of Ixtapaluca, Mexico, MX Mexico; 8 Autonomous University of Nuevo León, Mexico, NL Mexico; 9 Dr. Bernardo Sepúlveda Metropolitan Hospital, Mexico, NL Mexico; 10 Civil Hospitals of Guadalajara, Mexico, JA Mexico; 11 Celaya, Mexicali, MAC Hospitals Puebla, Mexico, PU Mexico; 12 Novo Nordisk Mexico, Mexico, DF Mexico; 13 Imperial College London, London, UK

**Keywords:** Obesity, Type 2 diabetes, Epidemiology

## Abstract

**Background:**

Patients with obesity have an increased risk for adverse COVID-19 outcomes. Body mass index (BMI) does not acknowledge the health burden associated this disease. The performance of the Edmonton Obesity Staging System (EOSS), a clinical classification tool that assesses obesity-related comorbidity, is compared with BMI, with respect to adverse COVID-19 outcomes.

**Methods:**

1071 patients were evaluated in 11 COVID-19 hospitals in Mexico. Patients were classified into EOSS stages. Adjusted risk factors for COVID-19 outcomes were calculated and survival analysis for mechanical ventilation and death was carried out according to EOSS stage and BMI category.

**Results:**

The risk for intubation was higher in patients with EOSS stages 2 and 4 (HR 1.42, 95% CI 1.02–1.97 and 2.78, 95% CI 1.83–4.24), and in patients with BMI classes II and III (HR 1.71, 95% CI 1.06–2.74, and 2.62, 95% CI 1.65–4.17). Mortality rates were significantly lower in patients with EOSS stages 0 and 1 (HR 0.62, 95% CI 0.42–0.92) and higher in patients with BMI class III (HR 1.58, 95% CI 1.03–2.42). In patients with a BMI ≥ 25 kg/m^2^, the risk for intubation increased with progressive EOSS stages. Only individuals in BMI class III showed an increased risk for intubation (HR 2.24, 95% CI 1.50–3.34). Mortality risk was increased in EOSS stages 2 and 4 compared to EOSS 0 and 1, and in patients with BMI class II and III, compared to patients with overweight.

**Conclusions:**

EOSS was associated with adverse COVID-19 outcomes, and it distinguished risks beyond BMI. Patients with overweight and obesity in EOSS stages 0 and 1 had a lower risk than patients with normal weight. BMI does not adequately reflect adipose tissue-associated disease, it is not ideal for guiding chronic-disease management.

## Introduction

Infection with the severe acute respiratory syndrome coronavirus 2 (SARS-CoV-2) and the resulting coronavirus disease-2019 (COVID-19) is a global pandemic that has collided with the on-going epidemic of chronic non transmissible diseases [1]. Although the majority of individuals infected with SARS-CoV-2 develop a mild disease, certain susceptible groups develop more serious forms, commonly characterised by respiratory failure [2]. Vulnerable groups include the elderly, immunocompromised individuals and those with pre-existing chronic diseases, such as arterial hypertension, diabetes, cardiovascular diseases, and respiratory diseases [3]. Many of these conditions coexist with obesity, and in younger individuals, obesity is a main promoter [4].

Obesity is increasing worldwide; with prevalence estimates of overweight and obesity in Mexico around 72% [5]. This is a chronic condition characterised by endocrine, metabolic and inflammatory disorders [6] that may influence the immune responses on exposure to SARS-CoV-2 [7]. Obesity is frequently present in patients requiring advanced medical treatment and those admitted to the ICU [8]. Several systematic reviews and meta-analyses have reported diverse associations of overweight and obesity with critical disease and mortality due to COVID-19 [9]. Rubio-Herrera et al. reported that obesity resulted in a 3.4-fold increased odds for developing severe infection [10].

Overweight and obesity are usually evaluated in clinical settings using body mass index (BMI); [11] however, despite the ease of use of such a parameter, it does not accurately reflect health status [12]. Anthropometric measurements do not reflect the presence or severity of obesity-related health risks, comorbidities, or functional limitations and are therefore inadequate for a comprehensive assessment of the complexity of obesity and to guide treatment interventions [13]. This has led to the development of clinical staging systems to assist in clinical decision-making of obesity treatments. Of these, the most practical and popular is the Edmonton Obesity Staging System (EOSS) which classifies the health burden associated with overweight and obesity into five stages based on a combination of patient’s medical, mental and functional disorders [13]. Numerous studies have confirmed the clinical utility of this staging system based on its prediction of complications following surgical and non-surgical weight loss, health service use, and mortality [14–17]. To date, however, BMI is the only obesity-related index to have been related to outcomes associated with COVID-19. We hypothesise that classification of overweight and obesity through EOSS will be better for predicting outcomes in these patients. The objective of this study is to evaluate in patients with normal weight, overweight and obesity hospitalised for COVID-19, the performance of the EOSS staging system compared to the traditional BMI classification, on COVID-19 outcomes (mechanical ventilation and death).

## Material and methods

Patients hospitalised for COVID-19 in 11 hospitals adapted as COVID-19 centres within Mexico’s National Health System. A database was created for this multi-centre cohort study and included all patients hospitalised with probable diagnosis (clinical features or compatible chest tomography) or confirmed diagnosis of COVID-19 by positive RNA PCR test between March 15 and May 15, 2020. Review of electronic and/or paper files and telephone calls were carried out by the research team to complete the required information. All participating hospitals provided IRB approval to review files and contact patients or their relatives to perform an EOSS questionnaire. Patients with incomplete data for the characterisation of EOSS and BMI categories were excluded from the analysis, so that finally the analysis was carried out in 1071 patients.

### Outcomes and variables

The variables included in the registry were: (1) clinical characteristics: age, sex, weight, height, BMI, smoking status, alcohol consumption, systolic and diastolic blood pressure, heart rate, respiratory rate, oxygen saturation on air (Sat O_2_), PaO_2_/FiO2 ratio, type 2 diabetes (T2DM), arterial hypertension (HTN), dyslipidaemia (DL), previous cardiovascular disease (CVD, cerebrovascular disease, coronary heart disease, heart failure and arrhythmias) and immunosuppression (IS), (2) biochemical parameters: haemoglobin, leucocytes, lymphocytes, platelets, C reactive protein, alanine aminotransaminase (ALT), aspartate aminotransaminase (AST), gamma glutamyl transferase (GGT), albumin, lactate dehydrogenase (LDH), glucose, blood urea nitrogen (BUN), creatinine and D-dimer, and (3) outcomes: critical disease (defined as respiratory failure requiring intubation, a ratio of the partial pressure of arterial oxygen to the fraction of inspired oxygen (PaO2:FiO2) <300 mmHg, and infiltrates in more than 50% of lung field [18], days from initial symptoms to admission, days from admission to hospital discharge, need for mechanical ventilation or admission to the ICU, days of mechanical ventilation and death.

### Determination of EOSS stage

**Fig. 1:**
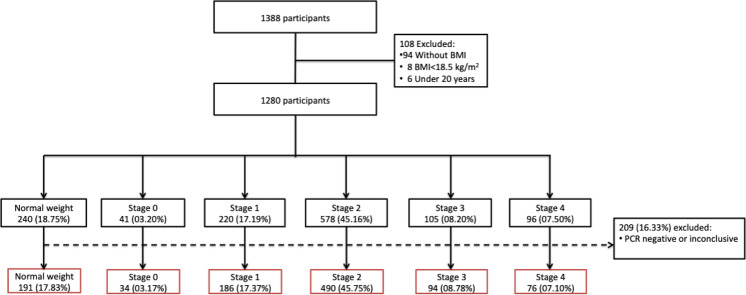
Flow diagram of patients included in the study. The patients were allocated in one of five stages according to their clinical features, and then reallocated after positive PCR result.

The EOSS classification was performed based on admission parameters of the COVID-19 registries of the participant hospitals in two phases. Firstly, patients who described themselves as healthy without previous diseases and pharmacological treatments, with normal parameters for arterial blood pressure, fasting glucose, total cholesterol, triglycerides, creatinine, and liver enzymes at admission were allocated to EOSS stage 0. Patients with systolic blood pressure between 130 and 139 mmHg and diastolic blood pressure between 80 and 90 mmHg, fasting glucose between 100 and 125 mg/dL, and/or HbA1c between 5.8 and 6.4% were allocated to EOSS stage 1; those who reported a previous diagnosis and/or pharmacological treatment for T2DM, HTN, DL and/or had blood pressure ≥140/90 mmHg, fasting glucose ≥126 mg/dL, non-fasting glucose ≥200 mg/dL, HbA1c ≥ 6·5%, total cholesterol >200 mg/dL, triglycerides >150 mg/dL and elevated liver enzymes but <2-fold the upper normal limit (UNL) were allocated to EOSS stage 2; patients who reported previous CVD, chronic renal or liver disease and/or clinical evidence of CVD, serum creatinine >1.12 mg/dL and liver enzymes ≥2-fold the UNL were allocated to EOSS stage 3; and patients with terminal cancer, CVD, kidney or liver disease were allocated to EOSS stage 4 (Fig. 1).

A questionnaire to determine the degree of functional impairment and assess mental health prior to hospitalisation was applied to patients through telephone calls after discharge or to a relative if the patient had succumbed to COVID-19. The questionnaire was adapted from the Edmonton Staging System description and has been used for the classification of patients in the Obesity and Eating Disorders Clinic programs at the National Institute of Health Sciences and Nutrition in Mexico City since 2015 (Supplementary Table 1) Investigators and co-investigators of all the collaborating centres delivered the survey. The principal investigator made contact with patients or relatives with major clinical complications, including death.

The results of this second phase, functional and mental health were added to the medical results. If one of these stages was superior to the initial EOSS medical allocation, the staging of the patient was changed to the highest staging obtained. For example, if a patient was initially allocated to EOSS 1 due to impaired fasting glucose but had major depressive disorder (mental health stage 2) and severe functional limitation (functionality stage 3), this patient would be considered to have EOSS stage 3. Due to a small number of patients in stage 0, these patients were grouped together with those of EOSS stage 1 (Supplementary Table 2).

### Statistical analysis

The clinical characteristics of the study population are presented as means (standard deviation, SD), median (interquartile range) or prevalence (%), as appropriate to the distribution of data. To identify specific risk factors for adverse outcomes in patients with a BMI ≥ 25 kg/m^2^, stratified analyses were carried out according to the following categories—normal weight (BMI < 25 kg/m^2^), overweight (BMI ≥ 25) kg/m^2^) and obesity (BMI ≥ 30 kg/m^2^). Comparison of clinical characteristics and biomarkers among patients with normal weight and within EOSS stages were analysed with ANOVA. Time-to-event survival analysis was carried out using Cox proportional risk regression models were generated to identify predictive risk factors associated with invasive mechanical ventilation and mortality, stratified by BMI categories and EOSS stages, and adjusted by age and sex. Multinomial logistic regression models were developed to explore the likelihood ratio of a patient with a positive COVID-19 test to be intubated or die. A confidence level of 95% was considered significant. The analysis was carried out with Stata version 15.

## Results

Of the 1071 patients included, all had complete data for classification of the medical EOSS staging and 787 (73%) had data for functional and mental EOSS staging. Table 1 shows the clinical characteristics and outcomes of patients with normal weight and patients with a BMI ≥ 25 kg/m^2^ within each EOSS category. Patients in EOSS stages 0–1 and 2 were younger and there was a greater proportion of men in all EOSS stages. BMI was similar among EOSS stages, only 7 to 8 kg/m^2^ higher than patients in the normal weight group. Patients with normal weight had a statistically lower prevalence of T2DM, HTN and kidney disease than patients with EOSS stages 2–4. Patients with EOSS stages 0–1 had the lowest values for fasting glucose, ferritin, D-dimer, higher oxygen saturation and the lowest prevalence of critical disease, mechanical ventilation and death.

**Table 1:** Baseline characteristics and outcomes according to EOSS stage.

Variable	Normal weight *N* = 191	Stage 0/1 *n* = 220	Stage 2 *n* = 490	Stage 3 *n* = 94	Stage 4 *n* = 76	*p*^a^
Age (yr), mean ± SD	57.1 ± 15.4	46.1 ± 12.7	51.4 ± 12.9	58.0 ± 14.7	58.4 ± 11.9	<0.001
Men, *N* (%)	134 (70.2)	161 (73.2)	302 (61.8)	58 (61.7)	51 (67.1)	0.024
BMI (kg/m^2^), mean ± SD	23.3 ± 1.5	30.6 ± 4.3	31.8 ± 6.2	31.5 ± 5.6	30.4 ± 4.7	<0.001
BMI categories, *N* (%)						
Overweight	–	122 (55.5)	228 (46.5)	49 (52.1)	40 (52.6)	0.148
Obesity	–	98 (44.6)	262 (53.5)	45 (47.9)	36 (47.4)	
Comorbidities, *N* (%)						
Type 2 diabetes	56 (29.3)	–	204 (41.6)	42 (44.7)	43 (56.6)	<0.001
Hypertension	54 (28.3)	–	167 (34.1)	49 (52.1)	39 (51.3)	<0.001
Asthma/COPD	5 (2.6)	5 (2.3)	17 (3.5)	3 (3.2)	3 (4.0)	0.902
Kidney disease	21 (11.0)	–	–	–	61 (80.3)	<0.001
Other relevant variables, *N* (%)						
Current smoking	30 (15.7)	44 (20.0)	92 (18.8)	10 (10.6)	14 (18.4)	0.452
NSAID	49 (25.7)	67 (30.5)	179 (36.5)	28 (29.8)	38 (50.0)	0.003
Antihypertensive treatment	47 (24.6)	–	139 (28.4)	39 (41.5)	30 (39.5)	<0.001
Biochemical and clinical parameters, mean ± DS						
Fasting glucose, mg/dL	166.4 ± 148.8	98.3 ± 14.5	176.4 ± 101.9	163.6 ± 80.5	179.0 ± 118.5	<0.001
Ferritin, mg/dL	918.0 ± 1124.5	791.3 ± 707.2	879.8 ± 1245.7	795.8 ± 747.1	1206.8 ± 1444.2	0.511
D-dimer, mg/dL	2384.0 ± 6633.9	1935.44 ± 5325.9	2511.70 ± 6963.0	2640.92 ± 6750.8	3571.2 ± 8217.3	0.063
Oxygen saturation, %	82.9 ± 12.5	85.4 ± 11.6	83.4 ± 12.7	80.5 ± 15.9	72.9 ± 20.7	0.011
Respiratory rate per minute	25.9 ± 9.2	25.2 ± 8.9	25.7 ± 8.1	26.37 ± 7.5	29.54 ± 15.5	0.019
Temperature, °C	36.9 ± 0.8	37.24 ± 0.9	37.2 ± 1.0	37.0 ± 0.8	37.0 ± 0.9	0.019
Severity of disease at admission for COVID-19, *N* (%)						
Critical	92 (48.2)	77 (35.0)	245 (50.0)	52 (55.3)	60 (79.0)	<0.001

^a^To evaluate within-group differences we performed chi square test, and ANOVA as appropriate. In case of non-parametric analysis, we used extension of the mean.

Compared to patients with normal weight, adjusted hazard ratios for mechanical ventilation in EOSS stages 2 and 4 were increased 42% (HR = 1.42, 95% CI 1.02–1.97) and 178% (HR = 2.78, 95% CI 1.83–4.24), respectively; whereas in BMI classes 35–39.9 kg/m^2^ and ≥40 kg/m^2^, HRs were increased by 71% (HR = 1.71, 95% CI 1.06–2.74) and 162% (HR = 2.62, 95% CI 1.65–4.17), respectively. Risk of death was 38% (HR = 1.38, 95% CI 0.42–0.92) lower in patients with EOSS stages 0 and 1 compared to patients with normal weight. A BMI ≥ 40 kg/m^2^ increased the risk of dying by 58% (HR = 1.58, 95% CI 1.03–2.42) (Table 2).

**Table 2:** Incidence of mechanical ventilation and case fatality in patients hospitalised for COVID-19 according to EOSS stages and BMI class, adjusted for age and sex.

	Mechanical ventilation		Death	
	Incidence (95% CI)^a^	HR^b^ (95% CI)	Incidence (95% CI)^a^	HR^b^ (95% CI)
EOSS				
Normal weight (<25 kg/m^2^)	2.53 (1.91–3.36)	Reference	3.05 (2.44–3.81)	Reference
Stage 0 and 1	2.04 (1.49–2.78)	0.73 (0.47–1.12)	1.63 (1.21–2.20)	0.62 (0.42–0.92)^†^
Stage 2	3.76 (3.21–4.40)	1.42 (1.02–1.97)^†^	2.58 (2.21–3.00)	0.96 (0.73–1.26)
Stage 3	3.69 (2.55–5.35)	1.30 (0.82–2.08)	2.33 (1.66–3.28)	0.86 (0.57–1.90)
Stage 4	7.92 (5.81–10.8)	2.78 (1.83–4.24)^‡^	3.42 (2.59–4.53)	1.33 (0.92–1.90)
BMI				
Normal weight (<25 kg/m^2^)	2.53 (1.91–3.36)	Reference	3.05 (2.44–3.81)	Reference
Overweight (BMI 25–29.9 kg/m^2^)	2.92 (2.44–3.49)	1.20 (0.86–1.68)	2.26 (1.92–2.66)	0.83 (0.63–1.10)
Obesity Class I (BMI 30–34.9 kg/m^2^)	3.84 (3.09–4.78)	1.37 (0.95–1.97)	2.40 (1.95–2.97)	0.95 (0.69–1.30)
Obesity Class II (BMI 35–39.9 kg/m^2^)	4.64 (3.22–6.68)	1.71 (1.06–2.74)^†^	3.16 (2.18–4.58)	1.26 (0.81–1.98)
Obesity Class III (BMI ≥ 40 kg/m^2^)	7.01 (4.96–9.92)	2.62 (1.65–4.17)^‡^	2.98 (2.13–4.17)	1.58 (1.03–2.42)^†^

Patients with BMI ≥ 25 kg/m^2^				
EOSS				
Stage 0 and 1	2.04 (1.49–2.78)	Reference	1.63 (1.21–2.20)	Reference
Stage 2	3.76 (3.21–4.40)	1.91 (1.35–2.72)^‡^	2.58 (2.21–3.00)	1.55 (1.10–2.19)^†^
Stage 3	3.69 (2.55–5.35)	1.74 (1.06–2.85)^†^	2.33 (1.66–3.28)	1.42 (0.89–2.26)
Stage 4	7.92 (5.81–10.8)	3.75 (2.38–5.90)^‡^	3.42 (2.59–4.53	2.19 (1.43–3.36)^‡^
BMI				
Overweight	2.92 (2.44–3.49)	Reference	2.26 (1.92–2.66)	Reference
Obesity Class I (BMI 30–34.9 kg/m^2^)	3.84 (3.09–4.78)	1.16 (0.87–1.54)	2.40 (1.95–2.97)	1.14 (0.87–1.49)
Obesity Class II (BMI 35–39.9 kg/m^2^)	4.64 (3.22–6.68)	1.46 (0.97–2.20)	3.16 (2.18–4.58)	1.52 (1.00–2.30)^†^
Obesity Class III (BMI ≥ 40 kg/m^2^)	7.01 (4.96–9.92)	2.24 (1.50–3.34)^‡^	2.98 (2.13–4.17)	1.92 (1.30–2.84)^‡^

^†^*p* < 0.05; ^‡^*p* < 0.001.

^a^Per 100 days/person.

^b^Adjusted for age and sex.

When restricting analysis to the group of patients with a BMI ≥ 25 kg/m^2^, the HRs for EOSS groups showed that, compared to stages 0 and 1, EOSS stages 2, 3 and 4 increased the risk for mechanical ventilation by 91% (HR = 1.91, 95% CI 1.35–2.72), 74% (HR = 1.74, 95% CI 1.35–2.72) and 275% (HR = 3.75, 95% CI 2.38–5.90), respectively. In contrast, assessment according to BMI group showed that the risk for mechanical ventilation was 124% (HR = 2.24, 95% CI 1.50–3.34) higher only in patients with BMI ≥ 40 kg/m^2^. With respect to mortality, EOSS stages 2 and 4 increased the risk of death by 55% (HR = 1.55, 95% CI 1.10–2.19) and 119% (HR = 2.29, 95% CI 1.43–3.36) compared to EOSS 0 and 1; whereas BMI 35–39.9 and ≥40 kg/m^2^ increased the risk of death by 52% (HR = 1.52, 95% CI 1.00–2.30) and 92% (HR = 1.92, 95% CI 1.30–2.84), respectively (Table 2).

**Fig. 2:**
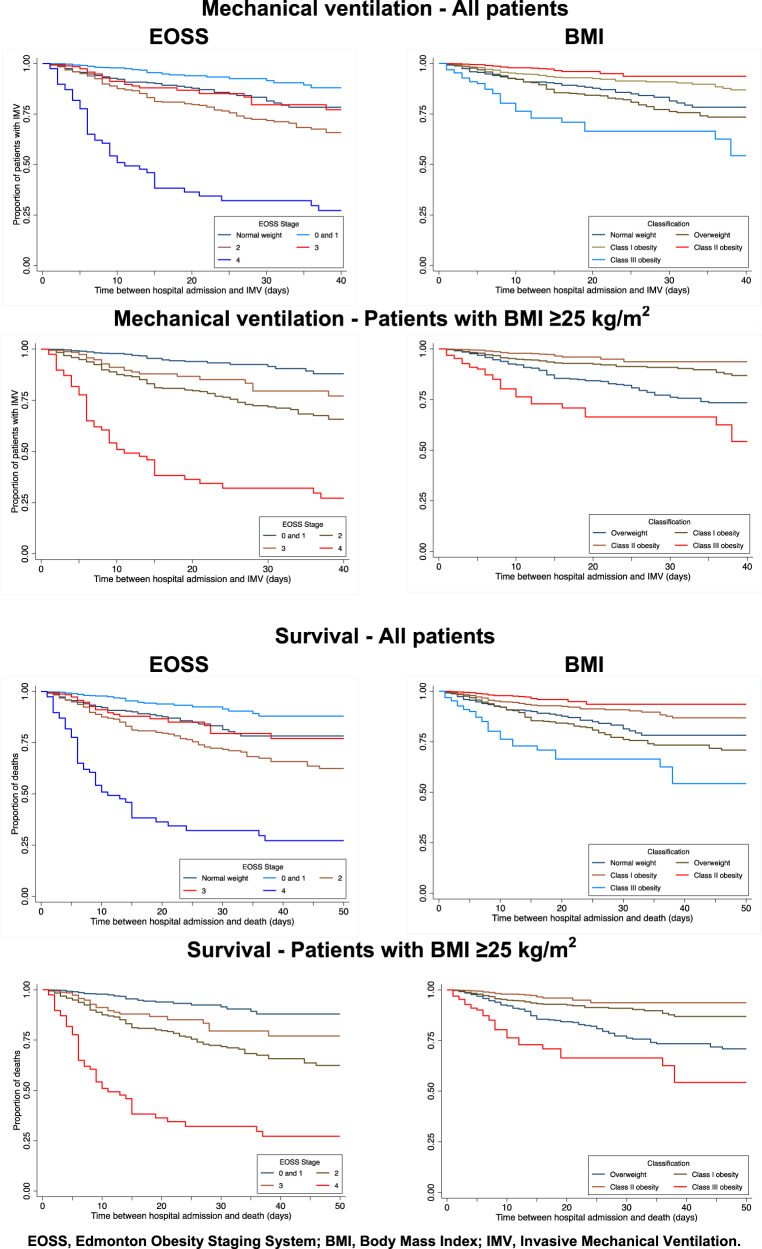
Survival analysis for invasive mechanical ventilation and mortality according to EOSS stage and to BMI classification. The graphs on the left show the results according the EOSS stage and those on the right according to the BMI for each of the items analyzed, adjusted for age and sex.

The overall survival analysis to evaluate time-to-invasive mechanical ventilation showed that EOSS stages 0 and 1 had the longest time-to-event, followed by patients with normal weight and patients in EOSS stage 2: EOSS stages 3 and 4 had significantly shorter time-to-mechanical ventilation. In contrast using the BMI classification, obesity classes I and II had the longest time-to-mechanical ventilation, followed by patients with normal weight and overweight; patients with class III obesity had significantly shorter time-to-event. Survival analysis of patients with BMI ≥ 25 kg/m^2^ showed reduced time-to-mechanical ventilation with increasing EOSS stages. In contrast, BMI showed the shortest time-to-event in patients with overweight and class III obesity (Fig. 2).

In terms of mortality, patients with EOSS stages 0 and 1 showed the highest survival, followed by patients with normal weight, EOSS stage 3 and 2 and EOSS 4. With respect to BMI, obesity classes I and II showed the longest survival, followed by normal weight and overweight; class III obesity had the lowest survival. Similarly, among patients with a BMI ≥ 25 kg/m^2^, those with EOSS stages 0 and 1 had the longest survival, compared to those with EOSS stage 4, who had significantly lower survival. Obesity classes I and II had longer survival than overweight patients, and the shortest survival time occurred in patients with class III obesity (Fig. 2).

The association of adverse outcomes with the EOSS staging was similar after combining EOSS stages into 0 + 1, 2, and 3 + 4 to reflect categories of (1) good health, (2) presence of established chronic condition(s) and (3) end-organ or terminal disease. According to these categories, risk for mechanical ventilation and death were increased by 84% and 237%, and 258% and 282% in EOSS stages 2 and 3 + 4 versus 0 + 1, respectively (Supplementary Table 3). The survival analysis showed that EOSS 2, 3 and 4 showed a progressive need for mechanical ventilation and similarly reduced survival compared to stages 0 and 1 (Supplementary Fig. 1).

## Discussion

The COVID-19 pandemic has had significant effects on physical and mental health and has highlighted the impact of chronic diseases on the complications of infectious conditions. The interaction of COVID-19 with the epidemic of modern life, obesity [19], is examined in this multi-centre study including diverse settings across Mexico. We examined the risk of severe COVID-19-related outcomes across different stages of BMI and the EOSS. Increasing BMI and EOSS stages were differentially associated with need for mechanical ventilation and mortality. These associations were largely attributed to patients in EOSS stages 3 and 4, characterised by advanced comorbid conditions and in those with class III obesity (BMI ≥ 40 kg/m^2^), with the lower obesity categories being relatively unrelated to COVID-19 outcomes and in absence of comorbid conditions. This highlights the importance of distinguishing healthy vs. comorbid in patients with overweight and obesity for assessing COVID-19 risk and interventions.

Of special consideration, the risks for adverse outcomes in patients with a BMI ≤ 25 kg/m^2^, (considered as ‘healthy’) are compared to those of patients with a BMI ≥ 25 kg/m^2^, categorised into different EOSS stages. Patients with normal weight, despite having a lower prevalence of T2DM, HTN and kidney disease, had similar risks for mechanical ventilation and death as patients with overweight and obesity in EOSS stages 2, 3 and 4 (established comorbidities, end-organ damage, and end-stage comorbidities). There was an increasing need for mechanical ventilation and death with progressive EOSS categories. In contrast, BMI predicted higher risk for adverse outcomes showed in patients with normal weight and overweight than those with class I and II obesity. Patients with normal weight only showed a lower risk for mechanical ventilation compared to class III obesity, consistent to epidemiological studies that show increased date rates in the highest BMI categories [20]. Moreover, patients with EOSS stages 0 and 1 had a lower HR for death than normal-weight patients.

Over the past two decades, many studies have found that BMI and other anthropometric measurements are unreliable target indices for effective interventions to reduce morbidity and mortality [15, 21]. Retrospective and prospective clinical and population studies have found differing associations between increasing BMI and adverse health outcomes, and certain studies have reported reduced CVD and mortality in individuals with overweight and mild to moderate obesity [22–25]. Thus, researchers have recommended that quality and function of adipose tissue is considered more important in clinical practice to determine overall health and CVD risk than the total amount of adipose tissue [12].

Variable associations have also been found between BMI and COVID-19 outcomes. A systematic review by Malik et al. reported an increased prevalence of COVID-19 and adverse outcomes in individuals with a BMI ≥ 25 kg/m^2^ and age >50 years in heterogeneous populations [26]. In contrast, data obtained from Kaiser Permanente reported that the association between COVID-19 mortality and obesity is stronger in younger patients [27]. In another systematic review, worse COVID-19 outcomes were associated with increasing BMI, starting from ≥25 kg/m², with prevalence rates varying from 13.3 to 68.6%. Increased risks were related to worsening of infection, increased prevalence of hospitalisations, worse outcomes, and greater mortality, especially in elderly patients with other chronic conditions [28]. Tamara and Tahapary [9] reported the consistent contribution of obesity as a risk factor for the requirement for invasive mechanical ventilation and mortality in patients with BMI greater than 35 kg/m^2^. A recent study in a single hospital in France reported worse outcomes in patients with a BMI ≥ 25 kg/m^2^, but similar mortality risks across all overweight and obese categories. There were significant differences in the frequency of comorbid conditions among the BMI categories that likely explain this result [29]. Therefore, the association between BMI and COVID-19 outcomes is variable and most studies report that this association is significant over a BMI of 35 kg/m^2^, and even stronger above 40 kg/m^2^ [30].

BMI, as a parameter of an individual’s size, does not accurately reflect the pathophysiological mechanisms of obesity, such as inflammation and metabolic disturbances. Evaluation of such mechanisms provides a more complete understanding of risks; for instance, in the case of hyperglycaemia, this has been shown to influence COVID-19 severity through production of reactive oxygen species and dysregulated immune response, along with glycaemic deterioration as a complication of COVID-19 [31, 32]. Chiappetta et al. have explored the use of EOSS in the setting of COVID-19 patients with obesity. They confirmed an association between increased levels of IL-6 and CRP with EOSS 2 and 3, without a significant association with BMI [33].

Furthermore, in patients with normal weight, prevalence of other chronic diseases that could have driven outcomes was not higher. Several studies have reported that normal-weight patients can have detrimental pathophysiological mechanisms, including poor quality of diet and low fitness levels, commonly considered to occur only in individuals with overweight and obesity [34, 35]. It is crucial to understand that the prioritisation of BMI not only incorrectly qualifies health status, but also misguides the optimal implementation of interventions to reduce risks and disease.

Although medical, functional, and mental health status are known to mediate health outcomes beyond traditional anthropometric measurements, (BMI, and sometimes waist circumference) these often remain the sole aspects that guide the treatment of individuals with overweight and obesity around the globe. BMI does not translate into a better understanding of the complexity of obesity [36]. As much as the timely management of people suffering with obesity is needed, it is also essential to move away from weight loss paradigms towards treatment based on the individual pathophysiology and needs of persons with obesity. Viewing obesity in terms of the clinical manifestations of its disorders will improve treatment of this disease. This study has strengths and limitations. This is the first study, to our knowledge, to assess the association of EOSS with COVID-19 outcomes in patients from multiple specialised centres standardising an EOSS classification. Among the limitations, this study had a retrospective design including patients under contingency-care. Height and weight were sometimes self-reported on interview. The functional and mental EOSS categories were not assessed in all patients due to incomplete response to the telephone questionnaire. Urgent care did not allow a complete determination of end-organ damage in all patients; this may have meant that some patients were allocated an EOSS stage 2 instead of stage 3, potentially explaining a lack of HR gradient in some analyses. Another limitation is that the questionnaires to determine functional and mental status were carried out by telephone after discharge. The outcome, whether the patient survived or not, may have affected their answers. However, several pragmatic studies that have assessed the utility of EOSS from registries and databases have employed such qualitative approximate information to establish the EOSS stages [14, 16, 37, 38]. Prospective studies with more accurate determination of EOSS are needed to confirm its utility in such clinical settings. Finally, other data that could influence mortality rates from COVID-19, such as socioeconomic factors and standards of care in each hospital was not available.

## Conclusions

In this multi-centre study, the categorisation of patients with normal weight, overweight and obesity using a clinical scheme such as EOSS to determine presence of comorbidity had adequate performance to predict adverse outcomes. In particular, patients in stages 0 and 1, despite being classified with overweight or obesity, had significantly lower mortality rates than patients in the normal-weight category.

The classification of obesity according to BMI may reflect risks associated with mechanical strain, and thus show higher risk for mechanical ventilation only at higher BMI values. However, an individual’s size does not reflect the pathophysiological mechanisms of adipose tissue dysfunction. Therefore, BMI is probably inadequate to guide chronic-disease management. This change in perspective may improve the future management of the obesity epidemic.

## Supplementary information

Supplemental Figure 1

Supplemental table 1

Supplemental table 2

Supplemental table 3

Authors Contributions
