# Supplementary material for: The utility of the Edmonton Obesity Staging System for the prediction of COVID-19 outcomes: a multi-centre study
Source: Int J Obes (Lond). 2022 Jan 1;46(3):661–8. doi: 10.1038/s41366-021-01017-8 (PMC8873002; doi:10.1038/s41366-021-01017-8)
Supplement: Supplementary file 1 — Supplemental Figure 1 [file 41366_2021_1017_MOESM1_ESM.docx]

Mexico City, October 15^th^, 2021

Dear Editors,

On behalf of my colleagues, please find enclosed the manuscript: “**The utility of the Edmonton Obesity Staging System for the prediction of COVID-19 outcomes: A multi-centre study**” which we hope may be considered for publication in the International Journal of Obesity.

The study aims to describe the predictive performance of the Edmonton Obesity Staging System (EOSS) for severe COVID-19 outcomes, highlighting the lack of BMI to consider the health status of the individuals categorized as overweight or with obesity.

In this multi-centre study, BMI was associated with adverse outcomes in its highest ranges, limiting its utility in the majority of population considered overweight and with obesity, who are within the ranges of a body mass index between 25-35.9 kg/m^2^. Despite that we did not have complete information to characterize all patients within stage EOSS stage 3, this scheme had increased risk for COVID-19 outcomes with increasing stages, even showing lower risk in patients with EOSS stages 0 and 1 with overweight and obesity compared to patients with normal weight in general.

The most important finding in the study was the highlight of possibility of BMI to wrongly classify individuals as “healthy” based on their size alone.

**Authors:**

- Dr. Marcela Rodríguez Flores (Corresponding author)
- Dr.Eduardo Wilfrido Goicoechea Turcott
- Dr.Leonardo Mancillas Adame
- Dr. Nayely Garibay Nieto
- Dr. Malaquías López Cervantes
- Dr. Mario E. Rojas Russell
- Dr. Lilia V. Castro Porras
- Dr. Eduardo Gutiérrez-León
- Dr. Luis Fernando Campos Calderón
- Dr. Karen Pedraza Escudero
- Dr. Karina Aguilar Cuarto
- Dr. Eréndira Villanueva Ortega
- Dr. Joselin Hernández Ruíz
- Dr. Guadalupe Guerrero Avendaño
- Dr. Sheyla Marcela Monzalvo Reyes
- Dr. Rafael García Rascón
- Dr. Israel Nayensei Gil Velázquez
- Dr. Dora Elia Cortés Hernández
- Dr. Marcela Granados Shiroma
- Dr. Brenda Giselle Alvarez Rodríguez
- Dr. Martha Leticia Cabello Garza
- Dr. Zaira Lucía González Contreras
- Dr. Esteban Picazzo Palencia
- Dr. Juana María Cerda Arteaga
- Dr. Héctor Raúl Pérez Gómez
- Dr. Roberto Calva y Rodríguez
- Dr. Gerardo Sánchez Rodríguez
- Dr. Leslie Denise Carpio Vazquez
- Dr. María Alexandra Dávalos Herrera
- Dr. Karla María Villatoro de Pleitez
- Dr. Melissa Daniela Suárez López
- Dr. María Guadalupe Nevárez Carrillo
- Dr. Karina Pérez Alcántara
- Dr. Roopa Mehta. (Corresponding author)
- Dr. Edurne Sandoval Diez
- Dr. Edward W Gregg

**Author Contributions:**

| ^·^Dr. Marcela Rodríguez Flores  (Corresponding author) | Study conceptualisation, data collection, funding acquisition, methodology, project administration, writing of original draft, writing of the manuscript, reviewing and editing of the manuscript |
| --- | --- |
| ^·^ Dr.Eduardo Wilfrido Goicoechea Turcott | Data collection, writing of the manuscript, reviewing and editing of the manuscript |
| ^·^ Dr.Leonardo Mancillas Adame | Study conceptualisation, data collection, methodology, writing of the manuscript, reviewing and editing of the manuscript |
| ^·^ Dr. Nayely Garibay Nieto | Data collection, data analysis, methodology, writing of the manuscript, reviewing and editing of the manuscript |
| ^·^ Dr. Malaquías López Cervantes | Study conceptualisation, data curation and analysis, methodology, reviewing and editing of the manuscript |
| ^·^ Dr. Mario E. Rojas Russell | Study conceptualisation, data curation and analysis, methodology, writing of the manuscript, reviewing and editing of the manuscript |
| ^·^ Dr. Lilia V. Castro Porras | Data curation and analysis, writing of the manuscript, reviewing and editing of the manuscript |
| ^·^ Dr. Eduardo Gutiérrez-León | Data collection, reviewing and editing of the manuscript |
| ^·^ Dr. Luis Fernando Campos Calderón | Data collection, reviewing and editing of the manuscript |
| ^·^ Dr. Karen Pedraza Escudero | Data collection, reviewing and editing of the manuscript |
| ^·^ Dr. Karina Aguilar Cuarto | Data collection, reviewing and editing of the manuscript |
| ^·^ Dr. Eréndira Villanueva Ortega | Data curation and analysis, methodology, reviewing and editing of the manuscript |
| ^·^ Dr. Joselin Hernández Ruíz | Data collection, reviewing and editing of the manuscript |
| ^·^ Dr. Guadalupe Guerrero Avendaño | Data collection, reviewing and editing of the manuscript |
| ^·^ Dr. Sheyla Marcela Monzalvo Reyes | Data collection, reviewing and editing of the manuscript |
| ^·^ Dr. Rafael García Rascón | Data collection, reviewing and editing of the manuscript |
| ^·^ Dr. Israel Nayensei Gil Velázquez | Data collection, reviewing and editing of the manuscript |
| ^·^ Dr. Dora Elia Cortés Hernández | Data collection, reviewing and editing of the manuscript |
| ^·^ Dr. Marcela Granados Shiroma | Data collection, reviewing and editing of the manuscript |
| ^·^ Dr. Brenda Giselle Alvarez Rodríguez | Data collection, reviewing and editing of the manuscript |
| ^·^ Dr. Martha Leticia Cabello Garza | Data collection, reviewing and editing of the manuscript |
| ^·^ Dr. Zaira Lucía González Contreras | Data collection, reviewing and editing of the manuscript |
| ^·^ Dr. Esteban Picazzo Palencia | Data collection, reviewing and editing of the manuscript |
| ^·^ Dr. Juana María Cerda Arteaga | Data collection, reviewing and editing of the manuscript |
| ^·^ Dr. Héctor Raúl Pérez Gómez | Data collection, reviewing and editing of the manuscript |
| ^·^ Dr. Roberto Calva y Rodríguez | Data collection, reviewing and editing of the manuscript |
| ^·^ Dr. Gerardo Sánchez Rodríguez | Data collection, reviewing and editing of the manuscript |
| ^·^ Dr. Leslie Denise Carpio Vazquez | Data collection, reviewing and editing of the manuscript |
| ^·^ Dr. María Alexandra Dávalos Herrera | Data collection, reviewing and editing of the manuscript |
| ^·^ Dr. Karla María Villatoro de Pleitez | Data collection, reviewing and editing of the manuscript |
| ^·^ Dr. Melissa Daniela Suárez López | Data collection, reviewing and editing of the manuscript |
| ^·^ Dr. María Guadalupe Nevárez Carrillo | Data collection, reviewing and editing of the manuscript |
| ^·^ Dr. Karina Pérez Alcántara | Data collection, reviewing and editing of the manuscript |
| ^·^ Dr. Roopa Mehta.  (Corresponding author) | Methodology, writing of the manuscript, reviewing and editing of the manuscript |
| ^·^ Dr. Edurne Sandoval Diez | Study conceptualisation, funding aquisition, project administration |
| ^·^ Dr. Edward W Gregg | Methodology, writing of the manuscript, reviewing and editing of the manuscript |

All authors have approved the manuscript and agree with its submission to International Journal of Obesity. We also declare that the article is an original research, and it has not been published or simultaneously submitted for publication, either partially or totally, by the authors themselves or any other authors. If the article is accepted, the authors transfer the copyright to International Journal of Obesity.

Contact information of corresponding authors is:

- Roopa Mehta: [roopamehta@yahoo.com](mailto:roopamehta@yahoo.com)

Metabolic Diseases Research Unit (UIEM), National Institute of Medical Sciences and Nutrition Salvador Zubirán, Vasco de Quiroga 15. CP 14080; Tlalpan, Distrito Federal, México. Phone: +52(55)54870900 ext 2407

We thank to you for considering our manuscript.

Sincerely,


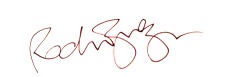


Roopa Mehta: [roopamehta@yahoo.com](mailto:roopamehta@yahoo.com)

Metabolic Diseases Research Unit (UIEM), National Institute of Medical Sciences and Nutrition Salvador Zubirán,
