# Supplementary material for: The utility of the Edmonton Obesity Staging System for the prediction of COVID-19 outcomes: a multi-centre study
Source: Int J Obes (Lond). 2022 Jan 1;46(3):661–8. doi: 10.1038/s41366-021-01017-8 (PMC8873002; doi:10.1038/s41366-021-01017-8)
Supplement: Supplementary file 2 — Supplemental table 1 [file 41366_2021_1017_MOESM2_ESM.docx]

**Supplemental table 1. Function and mental health questionnaire for EOSS staging in patients with a BMI ≥25 kg/m^2^**

Functionality (choose the number that applies to the patient): ______________________

| 0 | 1 | 2 | 3 | 4 |
| --- | --- | --- | --- | --- |
| Without limitation, can run | Good mobility. Some limitation to perform vigorous physical activity (unable to run, lift heavy weight), occasional pain or fatigue, shortness of breath with intense activity | Mild mobility impairment: Bending over, constant walking for 30 minutes, shortness of breath with moderate activity | Moderate disability: Limitation to climb a flight of stairs, constant walking for 15 minutes | Severe functional limitations: Use of a wheelchair, difficulty to walk one block, shortness of breath at rest |

Mental health (choose the number that applies to the patient): ________________

| 0 | 1 | 2 | 3 | 4 |
| --- | --- | --- | --- | --- |
| Without psychological symptoms | Psychological symptoms and/or mild deterioration of well-being, occasionally eating in response to stress | Moderate psychological symptoms: Anxiety, mild depression, binge eating once a week for ≥3 months | Significant psychological symptoms related to obesity: Panic attacks, depression with suicidal ideation | Severely disabling psychological/  psychiatric symptoms |
