# Supplementary material for: The utility of the Edmonton Obesity Staging System for the prediction of COVID-19 outcomes: a multi-centre study
Source: Int J Obes (Lond). 2022 Jan 1;46(3):661–8. doi: 10.1038/s41366-021-01017-8 (PMC8873002; doi:10.1038/s41366-021-01017-8)
Supplement: Supplementary file 3 — Supplemental table 2 [file 41366_2021_1017_MOESM3_ESM.docx]

**Supplemental table 2. Clinical characteristics of patients with normal weight versus patients with BMI ≥25 k/m^2^ and EOSS stages 0 and 1**

| **Variable** | **Normal weight (n=191)** | **EOSS stage 0 (n=34)** | **EOSS stage 1 (n=186)** |
| --- | --- | --- | --- |
| Mean age ± SD | 57.11 ±15.41 | 41.35 ±14.11 | 47.00 ±12.32 |
| Women N (%) | 57 (29.84) | 13 (38.24) | 46 (24.73) |
| Mean BMI ± DS | 23.29 ±1.52 | 30.67 ±3.83 | 30.61 ±4.40 |
| Current smoking N (%) | 30 (15.71) | 8 (23.53) | 36 (19.35) |
| Critical disease N (%) | 43 (22.51) | 3 (8.82) | 32 (17.20) |
| Previous NSAID use N (%) | 49 (25.65) | 8 (23.53) | 59 (31.72) |
| Death N (%) | 78 (40.84) | 4 (11.76) | 41 (22.04) |
