# Supplementary material for: The utility of the Edmonton Obesity Staging System for the prediction of COVID-19 outcomes: a multi-centre study
Source: Int J Obes (Lond). 2022 Jan 1;46(3):661–8. doi: 10.1038/s41366-021-01017-8 (PMC8873002; doi:10.1038/s41366-021-01017-8)
Supplement: Supplementary file 4 — Supplemental table 3 [file 41366_2021_1017_MOESM4_ESM.docx]

**Supplemental table 3. Incidence rates and Cox proportional hazard models adjusted for sex and age to predict mechanical ventilation and mortality in patients with BMI ≥25 kg/m^2^ and COVID-19 according to EOSS stages 0+1, 2, and 3+4**

|  | Mechanical ventilation | | Death | |
| --- | --- | --- | --- | --- |
|  | Incidence^¶^ (95% CI) | HR* (95%CI) | Incidence^¶^ (95% CI) | HR* (95%CI) |
|  | | | | |
| **EOSS** | | | | |
| Stage 0 and 1 | 2.08 (1.52-2.84) | Reference | 1.66 (1.23-2.24) | Reference |
| Stage 2 | 3.75 (3.21-4.39) | 1.84 (1.29-2.62)^‡^ | 2.58 (2.22-3.01) | 1.53 (1.09-2.15)^†^ |
| Stage 3 and 4 | 5.21 (4.11-6.59) | 2.37 (1.58-3.54)^‡^ | 2.82 (2.27-3.50) | 1.72 (1.17-2.52)^‡^ |

^¶^per 100 days/person; *Adjusted for age and sex; † p<0.05, ‡p<0.001
