# Supplementary material for: The utility of the Edmonton Obesity Staging System for the prediction of COVID-19 outcomes: a multi-centre study
Source: Int J Obes (Lond). 2022 Jan 1;46(3):661–8. doi: 10.1038/s41366-021-01017-8 (PMC8873002; doi:10.1038/s41366-021-01017-8)
Supplement: Supplementary file 5 — Authors Contributions [file 41366_2021_1017_MOESM5_ESM.docx]

**Supplemental figure 1. Survival analysis for invasive mechanical ventilation and death according to EOSS stages 0+1, 2, and 3+4 in patients with BMI ≥25 kg/m^2^, adjusted for age and sex**
